# Transition of clinical and basic studies on liver cirrhosis treatment using cells to seek the best treatment

**DOI:** 10.1186/s41232-021-00178-3

**Published:** 2021-09-16

**Authors:** Shuji Terai, Atsunori Tsuchiya, Yusuke Watanabe, Suguru Takeuchi

**Affiliations:** Division of Gastroenterology and Hepatology, Graduate School of Medical and Dental Sciences, Niigata University, 1-757 Asahimachi-dori, Chuo-ku, Niigata, 951-8510 Japan

**Keywords:** Liver, Cirrhosis, Fibrosis, Mesenchymal stromal cell, Macrophage, Exosome

## Abstract

The liver is a highly regenerative organ; however, its regeneration potential is reduced by chronic inflammation with fibrosis accumulation, leading to cirrhosis. With an aim to tackle liver cirrhosis, a life-threatening disease, trials of autologous bone marrow cell infusion (ABM*i*) therapy started in 2003. Clinical studies revealed that ABM*i* attenuated liver fibrosis and improved liver function in some patients; however, this therapy has some limitations such as the need of general anesthesia. Following ABM*i* therapy, studies have focused on specific cells such as mesenchymal stromal cells (MSCs) from a variety of tissues such as bone marrow, adipose tissue, and umbilical cord tissues. Particularly, studies have focused on gaining mechanistic insights into MSC distribution and effects on immune cells, especially macrophages. Several basic studies have reported the use of MSCs for liver cirrhosis models, while a number of clinical studies have used autologous and allogeneic MSCs; however, there are only a few reports on the obvious substantial effect of MSCs in clinical studies. Since then, studies have analyzed and identified the important signals or components in MSCs that regulate immune cells, such as macrophages, under cirrhotic conditions and have revealed that MSC-derived exosomes are key regulators. Researchers are still seeking the best approach and filling the gap between basic and clinical studies to treat liver cirrhosis. This paper highlights the timeline of basic and clinical studies analyzing ABM*i* and MSC therapies for cirrhosis and the scope for future studies and therapy.

## Introduction

**Fig. 1:**
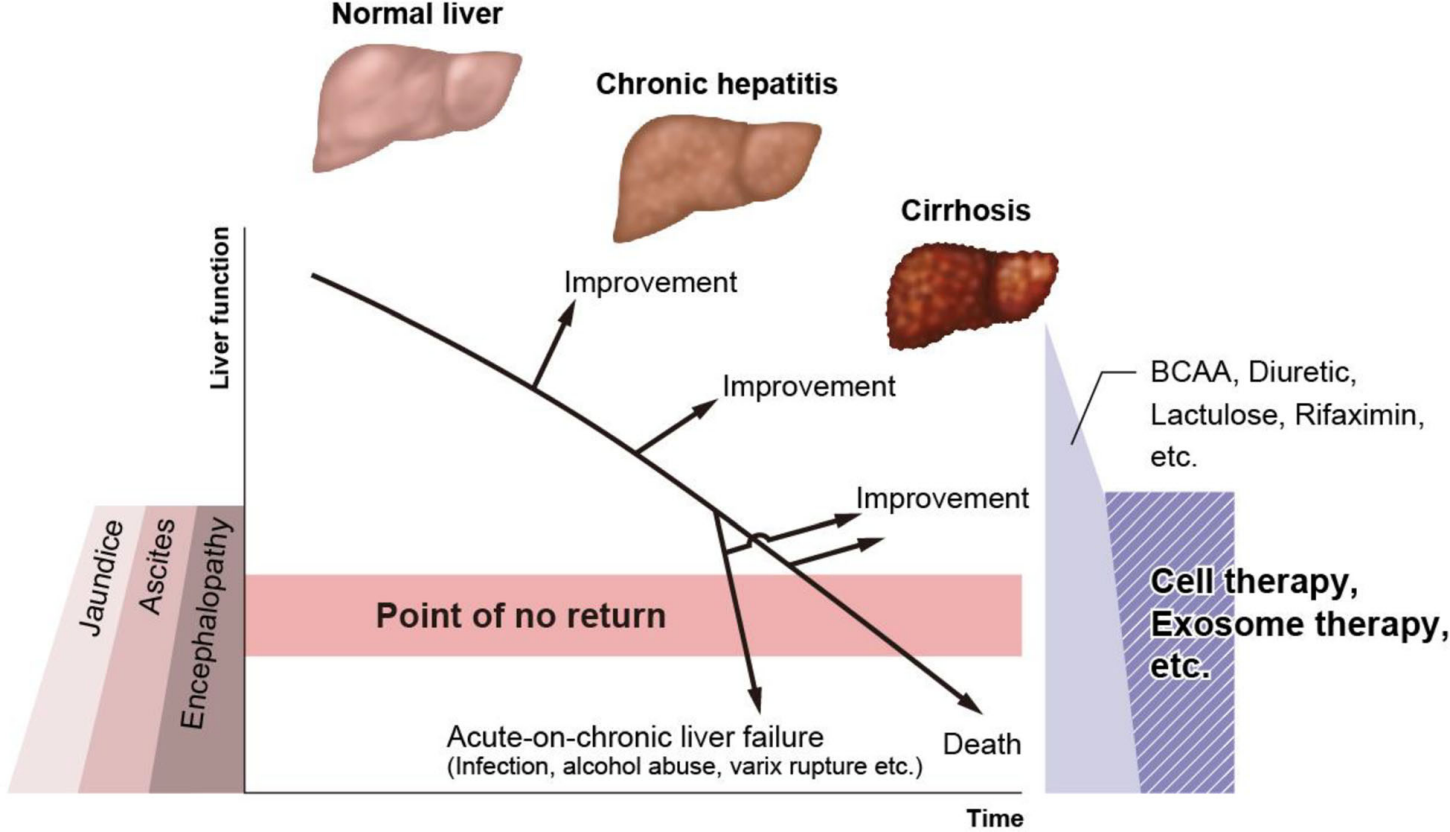
History of chronic liver disease, putative point of no return, and need for a new therapy

The liver is a highly regenerative organ, but long-term chronic inflammation followed by accumulation of fibrosis leads to cirrhosis. Generally, in compensated cirrhosis, the regenerative ability of the liver is retained when the damage caused by factors, such as hepatitis viruses and alcohol, is resolved; however, in decompensated cirrhosis, the regenerative ability weakens. Even if the causative factors are withdrawn, liver fibrosis sometimes worsens owing to infection or bleeding. This status is considered as a “point of no return” (Fig. 1); thus, early resolution of the causes of liver cirrhosis and regression of liver fibrosis are important to induce the ability of endogenous liver regeneration. To date, liver transplantation is considered the most effective therapy for cirrhosis; no other therapy has been approved for regressing liver fibrosis and for inducing endogenous liver regeneration [1–7].

Basic and clinical studies to develop a new treatment for cirrhosis are being performed vigorously. However, there are still gaps between basic and clinical study data in terms of therapeutic effects [8–10]. Here, the basic studies related to cirrhosis treatment using bone marrow cells, mesenchymal stromal cells (MSCs), and exosomes derived from MSCs are introduced [3, 11–18] and previous and ongoing clinical studies on cirrhosis [13, 19–21] are discussed with the prospect of developing effective therapies.

## Findings from basic studies

### Bone marrow cells

Approximately 20 years ago, an in vivo mouse model was developed to monitor the effects of administration of GFP-positive bone marrow cells in carbon tetrachloride (CCl_4_)-induced cirrhosis liver [11]. The intravenously administered cells migrated into the cirrhotic liver and attenuated liver fibrosis. This phenomenon is important because heterogeneous bone marrow cells include cells that can be used for regressing liver fibrosis. Furthermore, the repopulation of bone marrow-derived GFP-positive round-shaped hematopoietic-like cells expressing matrix metalloproteinase was identified in the damaged area of the liver [12]. However, the specific phenotype of these working cells attenuating liver fibrosis could not be identified.

### Mesenchymal stromal cells and macrophages

**Fig. 2:**
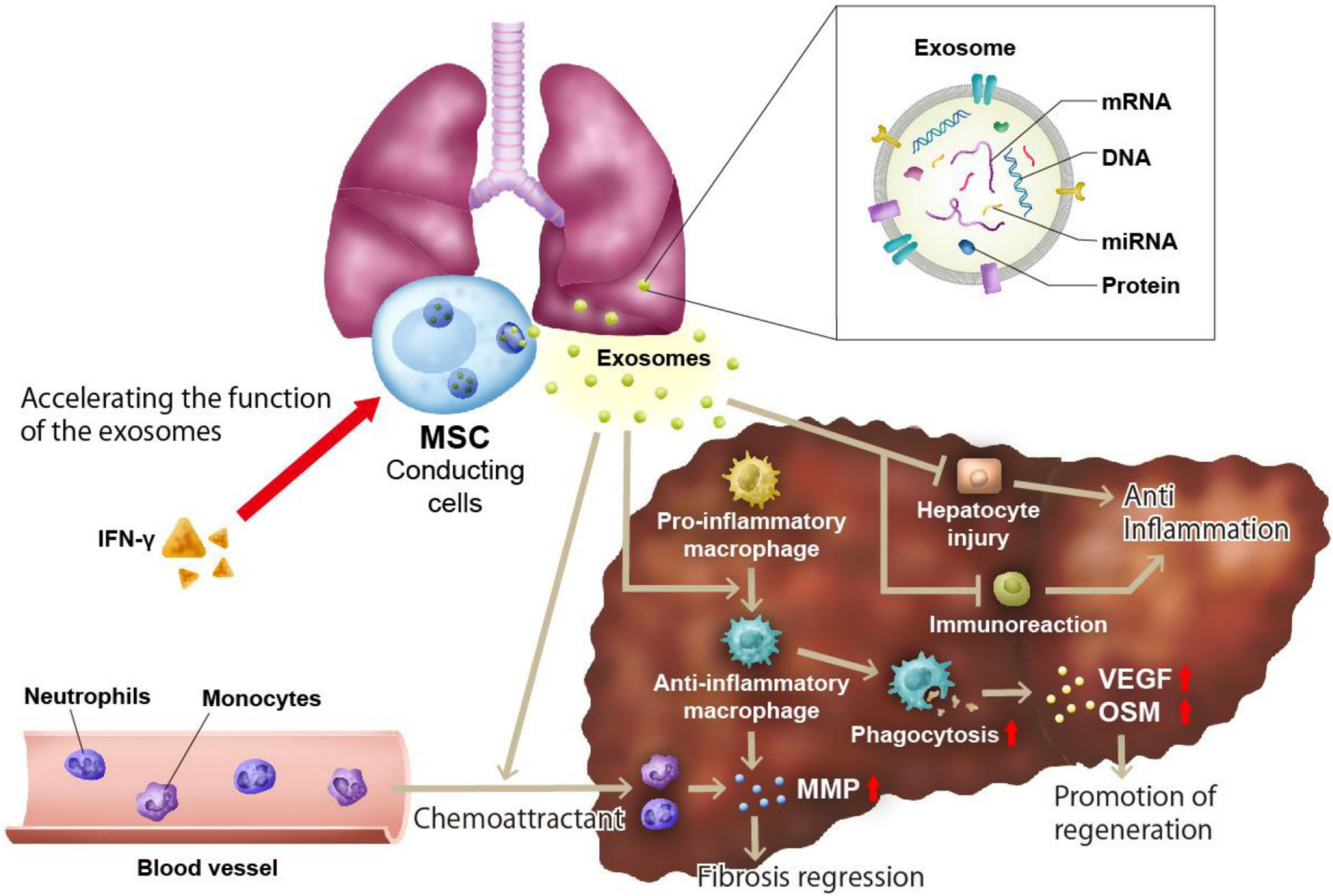
Results from the basic studies

As a next step, bone marrow cells crucial for attenuating liver fibrosis have been analyzed. The majority of the bone marrow cells are hematopoietic cells, whereas MSCs constitute a minor proportion. MSCs have garnered the attention of researchers worldwide as one of the candidate cells for the treatment of cirrhosis. The therapeutic effects of MSCs determined using animal models of cirrhosis have been reported [4]; however, gaining mechanisms of this therapy is difficult, requiring multi-directional analysis [22]. Some studies have reported that hepatocyte damage can be reduced by reducing inflammation or oxidative stress [23]. A few others have reported the effects of stellate cells [24]. There are several reports on the relationship between MSCs and macrophages [1, 5]. The relationship between bone marrow-derived MSCs and macrophages has been extensively evaluated. Some humoral factors from MSCs have been found to alter the polarity of macrophages into an anti-inflammatory phenotype. The therapeutic effects of bone marrow-derived MSCs and macrophages and the combination of MSCs and macrophages (total cell numbers were the same in each group) were evaluated. Unexpectedly, the combined therapy exerted the highest therapeutic effect on fibrosis. The distribution of MSCs and macrophages has also been analyzed in mice [16]. MSCs and macrophages were derived from Ds-Red transgenic and GFP transgenic mice, respectively. The GFP-positive bone marrow cell-derived macrophages and Ds-Red-positive MSCs were then injected into a mouse with CCl_4_-induced cirrhosis, and the distribution of these cells was analyzed in the liver, lung, and spleen by two-photon excitation microscopy. A small percentage of MSCs repopulated in the cirrhotic liver soon after injection, whereas most of the MSCs were trapped in the lung and disappeared after 7 days. On the contrary, many macrophages migrated into the damaged area of cirrhotic livers after injection for a week; some of them were phagocytizing hepatocyte debris. When hepatocyte debris was added to the macrophages, the macrophages started to produce oncostatin M and vascular endothelial growth factor, suggesting phagocytosis induces the regeneration of the liver [16]. From a series of results combined with previous observations, it is proposed that MSCs trapped in the lung function as “conducting cells,” attenuate liver damage and inflammation, and provide signals to “working cells,” namely, macrophages, to attenuate liver fibrosis (Fig. 2).

### Exosomes derived from mesenchymal stromal cells

Recently, in the analysis to identify key factors, exosomes secreted by MSCs have garnered attention [25]. Exosomes are extracellular vesicles of approximately 100 nm, acting as cargo for various proteins, miRNAs, and lipids for cell–cell communication [26]. Exosomes from MSCs are thought to be key factors involved in communications between MSCs and immune cells, especially macrophages. Exosomes after intravenous administration are mainly distributed in the liver and lung within 24 h and rapidly reduced thereafter [27]. Reportedly, macrophages are the main effector of exosomes [28]. On the basis of these findings, it may be ideal to use exosomes from MSCs for treating liver cirrhosis. Another attraction about exosomes is that they can be modified by pre-conditioning or gene editing of exosome-producing cells [29]. As MSCs are known to exert anti-inflammatory effects when inflammation in the host is high [30, 31], the use of MSCs pre-conditioned with cytokines or chemokines before harvesting the exosomes is an attractive strategy. For example, interferon-γ-preconditioned exosomes (γ-exosomes) could change the polarity of macrophages into an anti-inflammatory phenotype more effectively than exosomes without stimulation. γ-Exosomes also increase the migration and phagocytic abilities of macrophages. The characteristics of γ-exosomes were further evaluated in vitro using stellate cells; γ-exosomes did not inhibit the activation of stellate cells. The therapeutic effects of γ-exosomes were evaluated in the CCl_4_-induced cirrhosis model and their anti-inflammatory and anti-fibrotic effects were found to be superior to those of the MSCs and conventional MSC-derived exosomes. The effects of γ-exosomes in the CCl_4_-induced cirrhosis mouse were further evaluated by single-cell RNA-seq and two-photon excitation microscopy. γ-Exosomes were found to increase the frequency of anti-inflammatory macrophages in the liver and turn circular, increasing the number of macrophages that could contact the hepatocyte debris and migrate into the damaged area of the liver in a fixed time. The proteins and miRNAs were also compared between γ-exosomes and conventional exosomes, and it was found that they had changed after IFN-γ stimulation. Among these proteins, annexin-A1, lactotransferrin, and aminopeptidase N have been thought to play an important role in the polarization of macrophages [18]. From these findings, it could be concluded that MSCs can function as “conducting cells” through exosomes (especially γ-exosomes) by inducing the anti-inflammatory “working cells” (macrophages) and exert higher therapeutic effects in patients with cirrhosis (Fig. 2). Exosomes can be used as a stable drug delivery system.

## Clinical studies

### Autologous bone marrow cell infusion therapy

In parallel with the basic studies, a clinical study of cell therapy for decompensated cirrhosis was performed to overcome the “point of no return” [1]. First, autologous bone marrow cell infusion (ABM*i*) therapy was developed for patients with decompensated cirrhosis. For ABM*i* therapy, 400 ml of autologous bone marrow cells was harvested from the ilium; mononuclear cells were obtained and then infused from the peripheral vein. Owing to the need for general anesthesia, the criteria of ABM*i* therapy for patients with cirrhosis were as follows: serum total bilirubin under 3.0 mg/dl, PLT over 50,000/ml, and no obvious cardiovascular disease. The first human clinical study of ABM*i* therapy for patients with decompensated cirrhosis started in November 2003 and the treatment efficacy was reported in 2006 [19]. This study was expanded as a multi-center project for cirrhosis owing to the varying etiologies, including hepatitis B virus infections [20] and alcoholic related liver diseases [21]. Owing to the difficulties in evaluating the regression of fibrosis, at that time, some of the responders of ABM*i* therapy showed morphological improvement in the irregularity of cirrhosis, as observed in the computed tomography images, suggesting that ABM*i* therapy attenuates fibrosis [19]. In addition to improving the morphology, ABM*i* therapy decreased the serum granulocyte-colony stimulating factor and interleukin-1β levels and increased the number of proliferating cell nuclear antigen-positive proliferative hepatocytes [13] and cytokeratin 7-positive “liver progenitor cells” [20]. From these observations, it can be concluded that ABM*i* therapy attenuates liver fibrosis via sequential activation of liver regeneration.

### MSC therapy

While there were several responders to ABM*i* therapy, this therapy is relatively invasive and has some difficulties in spreading. Therefore, ABM*i* therapy has been shifted to autologous MSC therapy. Bone marrow cells, adipose tissue, and umbilical cord tissues are major sources of MSCs [22]. Some studies have reported the efficacy of autologous MSC therapy [8, 9]; however, the therapeutic effects appear to be restricted compared with those expected from the basic studies. It is well known that MSCs depict low immunogenicity; thus, allogenic MSC use for therapy without immunosuppressive agents is increasing in a variety of fields. Although adipose tissue is easily accessible during plastic surgery, harvesting allogenic bone marrow cells is difficult in Japan owing to regulations. Therefore, in the first Japanese trial using allogeneic MSC therapy, adipose tissue was selected as the source for allogeneic MSCs. Phase I/II clinical study was started in 2017 using allogenic adipose tissue-derived MSC therapy for decompensated cirrhosis with Rohto Pharmaceutical Co., Ltd. The study aimed to evaluate the safety and effectiveness of this therapy is ongoing. Compared to the favorable results from the basic studies, strong effects have not been observed in clinical studies. Researchers and clinicians are now seeking the optimal timing, disease stage, and cell types or culture conditions of cells to treat liver fibrosis effectively.

## Conclusions

**Fig. 3:**
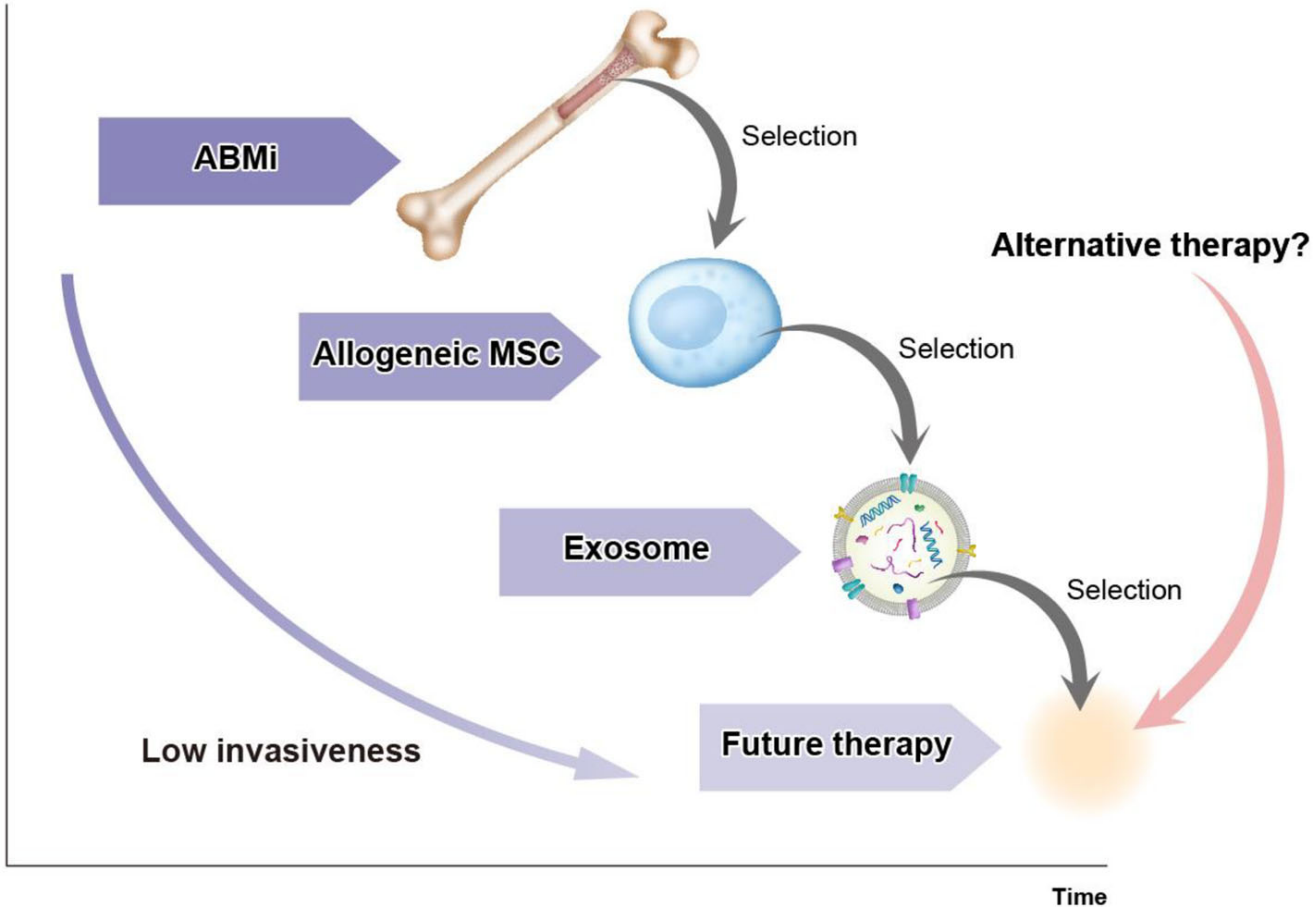
Evolution of cell therapy for cirrhosis and potential future therapy

Herein, the advances in basic studies using bone marrow cells, MSCs, and MSC-derived exosomes and clinical studies using autologous bone marrow cells and MSC therapy conducted in the past 20 years were discussed. Considering the basic studies, attenuating liver fibrosis and activating endogenous regenerative ability seem to induce anti-inflammatory “tissue repair” macrophages. The Forbes group of Edinburgh University reported phase I autologous macrophage therapy and its safety [32]. In addition to macrophages, the regulation of a variety of immune cells, including regulatory T cells, NK cells, and hepatic stellate cells, would be important to induce the activation of this endogenous regenerative ability [33]. Recent technological advances, such as two-photon excitation microscopy and single-cell RNA-Seq, have helped to demonstrate this phenomenon [18]. In addition, in the last two decades, researchers have focused on developing an evaluation tool for fibrosis. While invasive liver biopsy was the only tool for the accurate assessment of fibrosis, several non-invasive tools, such as serum markers, Mac-2 binding protein glycosylated isomer [34], autotaxin [35], and Pro-C3 [36], FibroScan [37], and elastography [38] using ultrasound sonography and magnetic resonance imaging, have been developed. It is important to determine markers or methods that are most efficient for the evaluation of fibrosis in clinical studies. These results can be used for the development of more effective therapy, induction of anti-inflammatory “tissue repair” macrophages, and evaluation of fibrosis and regeneration using the latest methods. At this point, exosomes are attractive candidates for next-generation cirrhosis therapy; however, promising alternative treatments can emerge (Fig. 3) [18, 39]. Continuous clinical and basic studies may be necessary for future therapy. A recent study has demonstrated that exosomes are an attractive alternative therapy for cirrhosis [18]. MSC-derived exosomes can be obtained from a variety of tissues, such as bone marrow cells, adipose tissues, umbilical cord tissues, and dental pulp. Theoretically, exosomes can be obtained from MSCs derived from induced pluripotent stem cells or embryonic stem cells. In addition, the contents of exosomes can be manipulated by pre-conditioning or gene editing, suggesting that more appropriate exosomes can be produced. However, exosome therapy is limited by the abundance of exosomes from cells and confirmation of uniformity of exosomes. However, new technologies and associated regulations to overcome these problems are expected. There are currently no Food and Drug Administration-approved exosome products, and the International Society for Cellular and Gene Therapies and the International Society for Extracellular Vesicles recognize the potential of extracellular vesicles (including exosomes) from MSCs and possibly other cell sources as treatments for several diseases [40]. Now, the Japanese Society for Regenerative Medicine has also formulated a working group to develop exosomes for the therapy. Other therapeutic cell-free strategies using peptides or oral drugs are also in progress [7]. In this milieu, cirrhosis treatment with cell and cell-free strategies has started to move to the next stages, allowing us to seek the best therapy.

## Data Availability

All data needed to evaluate the conclusions in the paper are provided in the paper. Additional data related to this study may be requested from the authors.

## Abbreviations

MSC: Mesenchymal stromal cell
CCl_4_: Carbon tetrachloride
ABM*i*: Autologous bone marrow cell infusion
